# Agent Based Models of Polymicrobial Biofilms and the Microbiome—A Review

**DOI:** 10.3390/microorganisms9020417

**Published:** 2021-02-17

**Authors:** Sherli Koshy-Chenthittayil, Linda Archambault, Dhananjai Senthilkumar, Reinhard Laubenbacher, Pedro Mendes, Anna Dongari-Bagtzoglou

**Affiliations:** 1 Center for Quantitative Medicine, University of Connecticut Health Center, Farmington, CT 06030, USA; koshychenthittayil@uchc.edu (S.K.-C.); larchambault@uchc.edu (L.A.); pmendes@uchc.edu (P.M.); 2 Department of Oral Health and Diagnostic Sciences, University of Connecticut Health Center, Farmington, CT 06030, USA; 3 Avon High School, Avon, CT 06001, USA; dhananjai284@gmail.com; 4 Department of Medicine, University of Florida, Gainesville, FL 32611, USA; reinhard.laubenbacher@medicine.ufl.edu; 5 Center for Cell Analysis and Modeling, Department of Cell Biology, University of Connecticut School of Medicine, Farmington, CT 06030, USA

**Keywords:** agent-based modeling, individual-based modeling, biofilm, microbiome, review

## Abstract

The human microbiome has been a focus of intense study in recent years. Most of the living organisms comprising the microbiome exist in the form of biofilms on mucosal surfaces lining our digestive, respiratory, and genito-urinary tracts. While health-associated microbiota contribute to digestion, provide essential nutrients, and protect us from pathogens, disturbances due to illness or medical interventions contribute to infections, some that can be fatal. Myriad biological processes influence the make-up of the microbiota, for example: growth, division, death, and production of extracellular polymers (EPS), and metabolites. Inter-species interactions include competition, inhibition, and symbiosis. Computational models are becoming widely used to better understand these interactions. Agent-based modeling is a particularly useful computational approach to implement the various complex interactions in microbial communities when appropriately combined with an experimental approach. In these models, each cell is represented as an autonomous agent with its own set of rules, with different rules for each species. In this review, we will discuss innovations in agent-based modeling of biofilms and the microbiota in the past five years from the biological and mathematical perspectives and discuss how agent-based models can be further utilized to enhance our comprehension of the complex world of polymicrobial biofilms and the microbiome.

## 1. Introduction

Challenges to the laboratory study of naturally occurring biofilms and host-associated microbiomes lie in the inherent complexity of these systems. The microbiota within biofilms form multi-species communities in which many different interactions (i.e., competition, antagonism, synergy and mutualism) may occur simultaneously among different species. A large number of species often coexist, which makes study of their potential interactions in the laboratory impractical. Many different micro-environments are encountered by microbes within biofilms on natural and artificial surfaces and in mammalian hosts. Conditions change over time as biofilms form, as digestive processes occur and as disruptions such as antibiotics or dietary changes are introduced. Such changes are difficult to predict or replicate in the lab. Under differing conditions, various individual cell characteristics and behaviors (phenotypes) may develop even within a single species of microbes. Such heterogeneity among individuals within a bacterial population allows bet-hedging, potentially allowing the population to better adapt to changing conditions. However, small local changes in conditions and the individual microbes’ response to them are difficult to measure using laboratory methods. The recent explosion in -omics techniques (genomics, proteomics, metabolomics, etc.) has created vast amounts of data that are difficult to validate with bench-top approaches. Modeling can generate hypotheses from these complex data sets that can then be validated experimentally. Many microbiome species have not yet been cultured but genetic and metabolic data collected through -omics approaches are beginning to be incorporated into ABMs, potentially allowing us to gain insights into the roles played by lesser-known species.

Mathematical modeling addresses some of the many challenges to the study of polymicrobial communities by mimicking complex environments computationally. Models can predict the outcome of many complex processes that are occurring simultaneously. Some models may utilize differential equations to understand the biofilm dynamics and structure, and others use optimization along with genomics and metabolomics [1,2,3,4]. In particular, the constraint-based reconstruction and analysis approach (COBRA) has been applied to human-microbial interactions [5].

**Figure 1:**
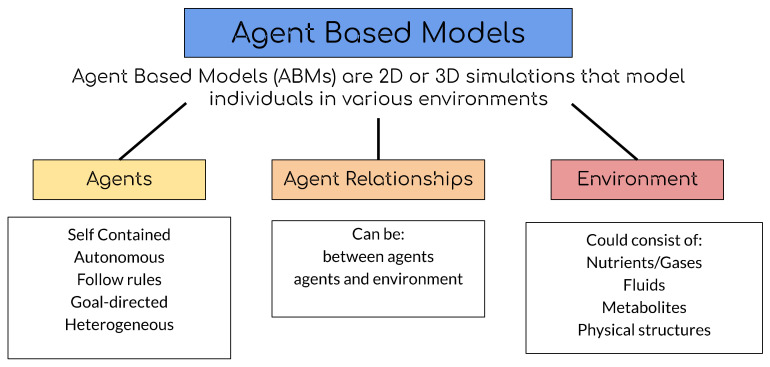
The description of agent-based models is based on Macal and North, 2005 [18].

One type of mathematical model is the agent-based model or individual-based model (ABM/IBM) (See Figure 1 for brief description). Some examples of the use of ABMs are in aiding policy and land-use management [6], developing conceptual models to understand social science issues [7,8,9], investigating the tumor microenvironment [10], and representing energy use in animal populations [11]. In microbiology, ABMs have been used to understand bacterial interaction in soil habitats [12], the pathogenesis of *Aspergillus fumigatus* infections [13], COVID-19 transmission [14], metabolic processes [15], and bacterial dynamics in experimental environments [16], including bioreactors [17]. Because they represent each microbe as an individual, these models are uniquely able to model interactions between individual microbes and between microbes and their environment. ABMs can help to explain emergent properties of microbial communities such as self-organized spatial patterns in biofilms.

In the intervening years since Hellweger and colleagues’ excellent 2016 review on this topic, the field has experienced an expansion in the application of ABMs to biofilm and microbiome studies [19]. The range of systems explored include industrial and medical optimization of biofilm growth, and exploration of the human microbiome. Models might focus on emergent biofilm structural characteristics or on the different phenotypes that develop in cells of the same species growing within a biofilm. The biological information used to build the model likewise covers a broad range. Inputs vary from extremely simple such as growth rates obtained from laboratory cultures, to very complex, incorporating entire genome-based metabolic networks, for example. Others have attempted to build extremely flexible and expandable models, able to incorporate many biological, chemical, and physical variables. Other recent reviews have focused on different computational methods for the microbiome and emerging priorities for microbiome research, which will not be addressed here [20,21,22].

In this review, we summarize the progress in agent-based modeling of biofilms and microbiomes over the past five years. In section two we present the biological questions investigated and summarize their results. The third section describes ABM techniques applied to the study of microbiomes and biofilms. We conclude with a discussion of the work done in the past five years and possible future research avenues.

## 2. Microbiological Questions Tackled by ABMs

### 2.1. Medical Microbiology

Microbes in the human body are found in biofilms on the skin or the mucosal surfaces of the digestive, reproductive and respiratory tracts. Biofilms are at the root of multiple medical problems. They grow on catheters and indwelling devices, which allows shedding of microbes into the bloodstream and systemic infection. Microbes in biofilms often become tolerant to antimicrobials, allowing them to persist on medical devices and in living tissues [23,24]. On the other hand, research is revealing an ever-expanding number of ways that our health depends on a well-balanced, commensal microbiome. Agent-based models have been used successfully to explore the many aspects of biofilms and the human microbiome that impact our health. Table 1 summarizes the various applications of ABM to microbial systems described below.

#### 2.1.1. Modeling of Biofilm Formation and Growth

Several models have been developed as flexible and accessible tools and researchers have subsequently adapted these models to explore specific biological questions. Other researchers have developed distinct models to address their specific research questions. Here we describe studies which investigate specific aspects of biofilm formation; some of the studies use models based on the open-source platforms iDynoMiCS and Netlogo, while others created models within proprietary software [47,48].

*Pseudomonas aeruginosa* forms biofilms on implanted and indwelling devices, and is a major cause of nosocomial infections such as ventilator-associated pneumonia and urinary tract infections [49]. Cystic fibrosis patients also suffer from chronic pulmonary *P. aeruginosa* biofilm infections, notoriously resistant to antibiotics [49,50]. Although bacterial cells in these biofilms are encased in an extracellular matrix that protects them from environmental dangers and immune system attack, detachment and dispersion of living cells is a well-characterized attribute of mature biofilms [51] that can lead to systemic infection and sepsis. In their adaptation of iDynoMiCS software, Li et al. explored the influence of 3 detachment mechanisms (shear, nutrient-limited and erosion) on *P. aeruginosa* biofilm structure [35]. Rather than model shear detachment using complex fluid dynamics, they made it a function of the thickness of the biofilm. Therefore, the biggest effects of shear detachment were seen in the later stages of biofilm growth. The same was true of nutrient-limited detachment, which didn’t affect the biofilm structure until it had become thick enough to limit nutrient diffusion. After this time, hollow areas developed in biofilm clusters and sloughing occurred. The erosion detachment mechanism was active throughout the time of growth and tended to create isolated clusters in the biofilm structure. The authors concluded that the mechanism of detachment affects the overall structure of the biofilm in a time-dependent manner and supported their modeling results with reports from experimental studies and previous modeling attempts [52].

Most experimental models of biofilm development start with adhesion of individual bacterial cells on a surface. However, in nature, bacteria often exist in aggregates of many cells, whereas single cells are rarer. Using well-coordinated ABM and experimental methods, Kragh and colleagues explored the role of cell aggregates in *P. aeruginosa* biofilm formation by modifying the iDynoMiCS software [34,47]. Their ABM was simple, incorporating only cell growth, competition for resources and mechanical interactions. Using their model and experimental biofilm formation in vitro, they tested various ratios of aggregates to single cells. When competition between aggregates and single cells is low, aggregated cells are at a growth disadvantage because cells in the center of the aggregate lack access to growth resources. However, when the density of single cells is high, single cell fitness is reduced due to competition. In this situation, aggregate cells have a relative fitness advantage because aggregate height allowed cells to reach growth resources not accessible to single cells. As might be expected, single cells formed a more uniform biofilm while aggregates contributed to a rough-textured biofilm. Addressing this biological question with an ABM allowed standardization of aggregate size and a degree of reproducibility which would have been practically impossible experimentally.

*P. aeruginosa* and *Candida albicans* are co-isolated from infections at several body sites, notably burn wounds and the lungs of cystic fibrosis patients [53]. These organisms influence each other’s virulence [54,55]. One mechanism proposed for this interaction involves a quorum sensing (QS) molecule produced by *P. aeruginosa* that inhibits the formation of tissue-damaging hyphal morphology in *C. albicans* [56]. A simple model by Pérez-Rodríguez and colleagues explores the effects of distance, distribution and orientation of the rod-shaped *P. aeruginosa* on the diffusion of QS molecules towards *C. albicans* yeast [39]. These parameters are difficult if not impossible to control experimentally. The model was calibrated using algebraic methods. The model can be adapted to other species or signaling molecules.

Dental caries, a highly prevalent disease worldwide, is associated with regular intake of refined sugars which drives a transition from a symbiotic to a dysbiotic makeup of dental plaque biofilms that, in turn, causes reduced pH, dissolution of enamel minerals, and caries lesions on tooth surfaces [32]. While frequency of sugar intake has been considered the primary factor leading to the selection of cariogenic bacteria, recent evidence points to an important role for total dietary sugar [57]. Head and colleagues adapted their previously created ABM to explore a large number of variations of frequency and total amount of sugar intake on plaque biofilm composition [58]. Total sugar strongly predicted the development of caries-associated dysbiotic biofilms along with low pH levels capable of dissolving tooth enamel. At very low and very high sugar intake levels, frequency of intake did not change the outcome. At intermediate sugar levels, the frequency of intake was a determinant in the formation of cariogenic plaque biofilms. These insights into the contributions of both amount and frequency of sugar intake can inform dietary guidance for improving oral health without the need for large-scale animal or human experiments.

*Helicobacter pylori* infects as many as 50% of humans worldwide and is the cause of peptic ulcers, gastritis and gastric cancer [59,60]. *H. pylori* forms biofilms in the mucosal lining of the stomach and its biofilm lifestyle may contribute to its ability to grow in the hostile gastric environment and to survive antibiotic treatment [59,61]. The quorum sensing (QS) molecule, autoinducer 2 (AI-2), acts as a chemorepellent in *H. pylori* and influences biofilm formation [62,63]. Sweeney, et al. adapted iDynoMiCS to explain differences in biofilm structure between wild type *H. pylori* and strains with mutations affecting their production, detection, or chemotaxis behavior to AI-2 [43,47]. Wild-type biofilms have a heterogeneous structure with towers and channels. Disruptions to their chemotaxis behavior results in thicker and more homogeneous biofilms while overexpression of AI-2 creates thinner and more heterogeneous structures. The overarching question they wished to investigate was whether biofilm structure is “a developmental program controlled by stage-specific gene expression” or “the outcome of local adaptations of individual cells”. More immediately, the biological question addressed by modeling, which was difficult to determine experimentally, was whether the structural differences emerged from the behavior of individual bacteria or from some other effect of the AI-2-related mutations. The authors adapted the existing iDynoMiCS modeling framework to include chemotaxis, monitoring of planktonic cells, and the ability of cells to join or leave the biofilm. Not only did the model determine that the chemotaxis behavior of individual bacteria could account for the differences in the biofilm structure, but it also brought to light an unexpected finding. While AI-2-dependent chemo-repulsion led to thinner biofilms, the number of cells joining the biofilm was actually larger in the AI-2 overexpression model than in the models of AI-2-deficient mutants. The authors explained that the more heterogeneous structure of over-expressor biofilms provided more surface area where planktonic cells were able to interact with biofilm cells. This behavior may have implications for treatment as increased interchange between planktonic and biofilm populations could introduce antibiotic-resistant or more virulent cells to the biofilm.

Structural features of biofilms such as “mounds”, “towers” and “mushrooms”, interspersed with channels are thought to enhance fitness through better access to nutrients, removal of waste and dispersal of quorum-sensing signals. Nontypable *Haemophilus influenzae* (NTHI) is a common causative agent of chronic and recurrent otitis media, found in biofilms on mucosal surfaces of the middle ear [64,65]. Extracellular DNA (eDNA) is a component of the EPS of many biofilms and several pathogenic roles have been proposed [66]. In a study using the methods of statistical physics to analyze high-resolution images of NTHI biofilms, Das and colleagues described the fractal nature of NTHI biofilms [29]. They hypothesized that eDNA could play a role in forming the fractal structures. They then created an ABM that simulated the production of eDNA and two effects it could have on the bacterial agents: Type IV pili (Tfp)-dependent twitching movement along a network of eDNA and a tendency for eDNA to prevent dispersion of agents into the surrounding media. This model was able to recapitulate the fractal nature of the NTHI biofilms and when eDNA was limited, the fractal structures failed to develop. Because eDNA release depends on the ComE pore of the NTHI outer membrane, a Δ*comE* mutant was used to confirm their predictions in experimental biofilms. This study nicely illustrates how ABMs can be used to test hypotheses through creation of rules, the results of which can then be tested using genetically altered organisms. One drawback to the study is that ComE is an essential part of the Tpf machinery so it was not possible to separate the roles of eDNA and twitching motility experimentally.

Biofilms are notoriously resistant to environmental stressors. One mechanism of this resistance is the formation of persisters, metabolically inactive cells that have a reversible tolerance to antibiotics and other stressors. Persister cells are produced both randomly and in response to environmental changes. The heterogeneity of environmental conditions within biofilms makes it difficult to study switching of cells between active and persister states under such conditions experimentally. A modeling project by Carvalho and colleagues aimed to predict how switching between susceptible and persister cell phenotypes could contribute to antibiotic resistance of biofilms [28]. Three switching strategies were modeled: constant, nutrient (substrate)-dependent and antibiotic-dependent. A constant switching strategy resulted in impaired fitness against antibiotic treatment, but substrate-dependent and antibiotic-dependent switching did not. If bacteria switch randomly into and out of a persister state, they will be killed by the antibiotic so long as the time of treatment is long enough for all to switch to a susceptible state. And of course, antibiotic-dependent switching led to excellent survival since persister cells only switched back to a vulnerable state once the antibiotic was removed. The fitness gained by substrate-dependent switching was perhaps not as intuitively predictable; low substrate-induced switching to the persister state did not greatly affect the overall growth rate of the biofilm since the cells in low-substrate areas would not have been contributing substantially to overall growth, even in the absence of antibiotic. However, the ability of those inner biofilm cells to enter a persister state allowed them to survive, then grow once the susceptible cells in the upper layers of the biofilm were killed, increasing the availability of substrate. Because of this dynamic, the longer period of antibiotic treatment was much more effective against bacteria that used substrate-dependent switching. The different spatial positioning of persister cells in the 3 strategies led to differences in biofilm structure which emerged during the recovery period. These modeling results have yet to be validated experimentally.

ABMs are an excellent tool for the exploration of evolution since they allow researchers to follow the fate of individuals subjected to varying conditions over time. Senescence is defined as the deleterious effects of accumulated damage. In unicellular organisms, damage could potentially be managed by repair or by asymmetric segregation of damaged cellular components when dividing. Wright and colleagues explored the question of which is the better strategy [45]. Previous studies considered this question only in planktonic states. While their previous study of cultures under constant conditions pointed to repair as the best strategy, the authors hypothesized that the clonal nature of biofilm populations and the heterogeneous conditions encountered by organisms in biofilms could favor a strategy of damage segregation over repair [67]. They created their model of a generic unicellular organism in the iDynoMiCS software, incorporating damage to proteins and a mechanism for cells to detect and respond to the damage. They used the model to test an adaptive repair strategy (that is, resources were allocated to repair in proportion to the level of damage) against asymmetrical segregation of damage (one daughter cell receives all damaged proteins upon division) under many conditions. The model predicted that repair is a better strategy under most conditions. Segregation of damage was only beneficial when repair was made less efficient or when nutrients were in extremely high concentrations. Their results were supported by a search of prokaryotic genomes which revealed that almost all contained at least one repair-related gene.

Understanding the early events of biofilm formation is critical to preventing harmful biofilms and encouraging growth of beneficial biofilms. Early ABMs modeled bacteria as spherical particles but physical constraints on other bacterial shapes may be quite different, a fact which has prompted modelers to incorporate another common bacterial shape: rods [68]. Acemel and colleagues created a model to study the earliest physical events of biofilm development: adherence, irreversible attachment and formation of microcolonies by a non-motile, rod-shaped bacterial agent [25]. They explored the effects of growth rates and simple Brownian motion on the structure of the developing biofilm in 2 dimensions. Physical parameters that predicted growth patterns included the aspect ratio (length/width) of the rod-shaped bacteria and the interplay between Brownian motion and growth. When growth dominated, tight microcolonies formed and growth occurred at the edges, but when cellular diffusion prevailed, colonies were dispersed, with growth occurring throughout a circular area, and filling in the spaces. Longer bacteria with high growth rates produced higher internal organization, with rods growing in clusters of similar orientation. Shorter rods formed less organized microcolonies with more random orientations of the particles. One aspect of the model, the Brownian diffusion rate, was cleverly manipulated experimentally to validate their predictions. The attachment-deficient, non-motile mutant, *Pseudomonas putida* MRB52, forms loose colony structure compared with the tight, highly organized colonies of the wild type, *P. putida* KT2442. As diffusion was reduced with increasing concentrations of dextran sulfate, *P. putida* MRB52 colonies grew progressively denser and more organized, matching the relationship between diffusion rate and colony architecture predicted by the model.

High-resolution imaging, made possible by recent advances in microscopy, is revealing structural features of biofilms at the level of the single-cell. *Vibrio cholerae*, the gastro-intestinal disease agent, forms biofilms with a high level of internal organization with rod-shaped organisms vertically ordered, especially in the center of expanding biofilms [69,70]. Beroz and colleagues created a simple agent-based model to explore the mechanical basis for this phenomenon [27]. In developing *V. cholerae* biofilms, cells grow and divide along their long axes, lying parallel to the surface. After several hours of growth, cells in the center of the expanding cluster begin to reorient to a vertical position. This reorientation spreads outward as the colony grows, maintaining a rough circle of vertical cells in the center with a ring of horizontal cells around it. The modelers were able to recreate this phenomenon by modeling forces acting on each cell: cell-to-surface adhesion and pressure exerted by neighboring cells in the x, y and z directions. They explored the effect of cell length on verticalization using both models and in vitro experiments in which chemicals altered the length of *V. cholerae* cells. Several similarities in modeled and experimental biofilms were noted: verticalization events were linked with cell division, and cell length had similar effects on the surface expansion and the overall shape of the developing biofilm. The study is a fine example of the use of modeling to learn how mechanical characteristics of individual cells can predict emergent features of biofilms.

Adhesion of microbes to a surface is the initial step in biofilm formation and has been intensely studied in that context [71]. The goal of a study by Schluter and colleagues was to investigate the evolutionary costs and benefits of adhesiveness within a growing biofilm [41]. They extended an existing ABM to include adhesiveness, affecting bacterial agents in two ways: by resisting removal from the biofilm through erosion and by resisting movement within the biofilm. While adhesion was necessary for cells to remain in a biofilm, it exacted a cost in loss of mobility, which was detrimental to highly adherent cells located at the base of a growing biofilm when nutrient concentrations were limiting. Less adherent cells were displaced upwards and had better access to nutrients supplied from above. These competitive dynamics changed when nutrients were either abundant throughout the biofilm or were supplied from below. The authors used their model to explore the role of EPS, which can influence adhesion and also serves to expand the volume occupied by cell clusters. When limiting nutrients were supplied from above, volume expansion by means of EPS production could compensate for the loss of fitness due to adhesion. When nutrients were provided from below, EPS was advantageous because it allowed cells to quickly colonize the substratum. Some predictions generated by the model were tested in fluorescence-expressing *V. cholerae* mutants with either constitutive EPS production or no EPS production. These experiments shed further light on the mechanisms involved in the competitive advantage of EPS production: EPS+ cells grew in lineage-related clumps and displaced EPS- cells from the substrate. The experimental data were used to make adjustments to the model which then produced results more consistent with the experiments. This study indicates the importance of adhesion to competition between microbes as they compete for space and nutrients in growing biofilms.

In another study addressing physical aspects of early biofilm events, Hartmann and colleagues describe biofilms as “self-replicating active liquid crystals” [31]. These researchers used automated confocal microscopy of growing biofilms to guide their creation of an agent-based model with the goal of learning whether the structure of *V. cholerae* biofilms could be predicted entirely on the mechanical interactions between individual cells. They compared their model predictions to actual biofilms of wild type *V. cholerae* and a *rbmA*-expression mutant in which the level of RbmA, a protein that mediates cell-cell adhesion, was controlled by arabinose levels. They concluded that physical interactions between cells can account for biofilm structure, at least in the early stages of *V. cholerae* biofilm formation.

#### 2.1.2. Agent Based Models of the Microbiome

One of the properties of the human microbiome that creates a challenge for experimental biologists and modelers is the remarkable number of different species that populate the human oral cavity and digestive tract. Estimates put the number of individual bacteria, archaea and single-cell eukaryotes in a human body at 3.8 × 10^13^, around the same order of magnitude as the number of human cells [72]. ABMs have been limited by the computational expense (time and computer capacity) of modeling large numbers of bacterial species. In addition, one goal of modeling is to create the simplest model that can adequately replicate important characteristics of a natural system. Because several species in the microbiome serve similar metabolic functions, one approach has been to model bacterial metabolic types rather than individual species. Another approach extends the ability of ABMs to include more species by carrying out computations in parallel. In this section, we describe four gut microbiome models in more detail.

Interactions between major gut bacterial types were modeled extensively by Shashkova and colleagues [42]. The focus of their work was the interactions that contribute to the maintenance of a healthy balance in the gut microbiota. Their ABM models only two bacterial types that represent the most numerous phyla in the gut, Firmicutes and Bacteroidetes, along with metabolites and the gut mucosa. The authors hypothesized that a stable modeled microbial community would develop only with metabolic feedback between the bacterial types. They tested numerous combinations of interactions (feeding, toxin/antitoxin) plus perturbation with antibiotics with the aim of finding the minimum number of interactions required to create and then re-establish a stable system after a disturbance. They discovered that one feedback mechanism was adequate to establish a stable state but that multiple feedbacks could result in more than one stable state. Feedback mechanisms also affected the spatial distribution of the bacteria in the gut, something they would not have discovered without a visual output as part of their model. The spatial arrangement of the bacteria controlled their ability to withstand antibiotic treatment as layering can protect those closest to the gut wall. Recovery from disturbance occurred more quickly when the microbiome contained a greater number of feedbacks. While questions such as these can be tested experimentally using bacterial strains genetically modified to interfere with certain interactions, the value of modeling is clear in its ability to test many hypothetical interactions and single out those that are likely to be important for further study in the laboratory.

Autism Spectrum Disorder (ASD) affects more than 1% of children in the U.S. and the origins of this disorder are not well understood. One hypothesis states that imbalances in pro-inflammatory (i.e., *Clostridia* and *Desulfovibrio*) and anti-inflammatory microbes (i.e., *Bifidobacteria*), lead to the development of ASD [73]. To test this hypothesis in silico, Weston and colleagues used existing information from the literature concerning direct and indirect interactions between these three bacterial genera to construct an ABM in an existing modeling framework, NetLogo [46,74]. Factors tested included the initial size of the bacterial populations, their growth rates, their competition for nutrients, and the effect of prebiotics and lysozyme. Decreases in the growth rates of the pro-inflammatory bacteria tipped the balance of the community towards a community composition associated with health, while increasing *Clostridium* growth rate or decreasing that of *Bifidobacteria*, created a steady state that theoretically favors ASD. Differences in the initial number of bacteria did not affect the eventual steady state reached by the population, but adding prebiotics gave an advantage to the anti-inflammatory *Bifidobacteria* that could compensate for a lower growth rate. Lysozyme treatment caused a large decrease in *Clostridia* and thus may reduce the potential for developing ASD.

Building on their previous work, this group created GutLogo, an ABM based on NetLogo, to model a community of four bacterial genera that are members of the human gut microbiota, adding *Bacteroides* to the three genera modeled previously [37,46]. The biological setting is the human ilium with six carbohydrates as nutrient sources. The model simulates population responses to changes in flow rate, nutrition and probiotics. The authors first adjusted the doubling times of the bacterial agents to achieve a steady state that matched data from the literature. They then introduced perturbations: probiotics (*Bifidobacteria*), changes in diet (2× higher or lower glucose), or changes in flow (constipation or diarrhea). Differences in glucose level altered the *Bifidobacteria* and *Desulfovibrio* populations; under high glucose, *Desulfovibrio* rose sharply but disappeared under lower glucose. Interestingly, adding a probiotic did not appreciably change the steady state levels of the four genera. Under constipation, all bacteria increased in numbers and all were reduced in numbers by diarrhea. The model was validated by comparisons with previously published experimental data.

The flexible modeling platform BacArena, described in detail in Section 3.1.4, was tested on a multispecies model of the gut microbial community [26]. Bauer, et al. based their model on SIHUMI, a seven-species SImplified HUman intestinal MIcrobiota, which was previously created in gnotobiotic rats to test the effects of changes in diet on bacterial cell counts and metabolism [75]. The ABM focused on metabolic interactions among bacterial species and between host and bacteria and how these interactions shape the spatial arrangements found within the gut microbiome. In the first iteration, metabolites for each of the 7 species were included. In this condition, the microbiome developed in a dysbiotic way, with *Escherichia coli* becoming dominant. However, when host production of mucus glycans was added to the model, the mucus layer on the gut wall became dominated by *Bacteroides thetaiotamicron*, a species which is able to degrade glycans, creating fermentation products that can be utilized by other species. Under these conditions the microbiome developed in a similar way to SIHUMI in gnotobiotic rats [75]. Thus, in this case, a previously published experimental study was used as the basis for changes to make the model more realistic.

In contrast to models treating the microbial members of the microbiome as individual agents, some ABMs address the microbiome as a property of the host who is modeled as an agent [76,77,78]. The neutral models of microbiome evolution by Zeng et al. consider how different host agents acquire their microbiomes and the role of the host or the environment in shaping the microbial community [76,77]. The microbiome is acquired via the parent, environment or both. Another such study describes VERA, an agent-based model created by Glushchenko et al., that focuses on propagation of antibiotic resistance in the host microbiota [78]. It uses gene transfer of resistance determinants within the community. The agents are hosts who are either healthy or infected and whose microbiota have different levels of antibiotic resistance. These models shed light on the role of the host in microbial evolution.

### 2.2. Industrial Microbiology

Microbes impact industry in negative and positive ways. Biofilms corrode equipment, increase fluid resistance in pipes, and drag forces on the hulls of ships, causing significant economic loss [79,80]. Biofilm formation on equipment and microbial contamination of foods are serious problems for the food-processing industry [81,82]. On the plus side, microbes are used in the production of chemicals and biofuels, in wastewater treatment, in fuel spill cleanup, and in preventing food spoilage [83]. ABMs are becoming popular design tools for industrial-scale biotechnological systems.

Synthetic biology researchers, Rudge and colleagues, created the ABM Cell Modeler with physical interactions and efficiency in mind [40]. They used their model to investigate the ways that cell shape and orientation affect biofilm structure. They were able to convincingly and quickly model several situations that had previously been explored experimentally. Researchers studying food contamination created an ABM to model growth of *E. coli* in biofilms on equipment and as micro-colonies submerged in a semi-solid food [44]. *E. coli* is a facultative anaerobe and adjusts its metabolism according to the available oxygen. Tack et al. used flux balance analysis (FBA) to model the complex *E. coli* metabolic network. In two case studies, they found that when low oxygen levels occur due to limitations to diffusion, cells within the community undergo metabolic differentiation and secrete weak acids; this causes local reduction in pH. Acid stress causes suppressed growth and cell death at the center of the submerged colonies and near the abiotic substrate in the biofilm case, which leads to detachment of the biofilm. In addition to metabolism, attributes of the model organisms included growth, death, division, shoving, adhesion to the substratum and each other, and detachment. These factors accounted for much of the *E. coli* biofilm characteristics highlighted in their references to previous experimental work.

Many models focus on either the biological, chemical or physical aspects of biofilm formation and growth but some researchers have attempted to accurately model all three. Jayathilake and colleagues developed an ABM containing 3 bacterial functional groups, extracellular polymeric substances (EPS) and inert cells [33]. They first tested the effect of nutrient gradients on biofilm formation. When gradients are less apparent, growth rate is faster and occurs throughout the biofilm, which is compact and smooth-surfaced. When nutrient gradients are greater, overall growth is slower, growth occurs mainly at the top of the biofilm, and a thicker, rough-surfaced biofilm forms. These results match those of other biofilm simulations. In a second set of trials, the authors modeled bacterial detachment under shear forces as an emergent property, a function of bacteria-EPS adhesion. The model replicated deformation of biofilms, formation of streamers and detachment of clusters. Flow affected the surface topography of the biofilms: under no shear they had very rough surfaces, with medium shear force they had undulating surfaces, and under high shear, smooth surfaces. Results are similar to those seen in experimental studies except that the model lacked sloughing of large sections of biofilms that takes place at later stages of growth.

More recent work from this group introduces the open-source software, Newcastle University Frontiers in Engineering Biology (NUFEB) [36]. Like its predecessor, it incorporates three-dimensional modeling of biological, chemical, and mechanistic properties of individual microbes in an ABM [33]. With this new version of their model, they repeated their study of a single-species biofilm growing in fluid flow, obtaining results similar to the previous version and matching well with experimental data. They again modeled the 3-bacteria biofilm community of heterotrophs (HET), ammonia oxidizing bacteria (AOB), and nitrite oxidizing bacteria (NOB). In this more complex model, new interactions emerged. The HET grew faster and dominated the biofilm at first, but as organic substrate was depleted, the nitrifying bacteria (AOB) took over. Surprisingly, the NOB didn’t grow, apparently because low O_2_ levels at the bottom of the biofilm were established by the time NO_2_^-^ was produced in sufficient amounts by the AOB.

The stated aim of a third recent paper from this group is to move ABMs from research tools to engineering tools which will require even larger and more physically and biologically precise modeling methods [30]. Because the laws of thermodynamics have predictive power in microbial communities, the authors altered NUFEB to incorporate a separate thermodynamics module which estimates overall biomass yield. The scenarios modeled are a 2-functional-group nitrification model (aerobic) and a three-functional-group anaerobic community. In the aerobic nitrification system containing AOB and NOB, they tested three scenarios related to pH-constant, freely changing, and buffered. They found differences in the dominant and limiting chemical species: with freely changing pH, NH_4_^+^ becomes the dominant form, NH_3_ becomes limiting and the AOB do poorly. However, when pH is buffered, O_2_ becomes limiting and the number of inert (dead) cells increases. Constant pH led to biofilms with NH_3_ and O_2_ becoming limiting in different areas. The anaerobic system contained glucose fermenters, acetoclastic methanogens and hydrogenotrophic methanogens. Because the model was modular, the authors were able to test it with and without thermodynamics coupled to the basic model. The two methods produced similar biofilms which served as a validation of the thermodynamics method. Overall, the incorporation of thermodynamics in ABMs has advantages in that it can be applied to any system in which the main redox reaction pairs are known and it relies on simpler factors unlike Monod kinetics. The consideration of pH effects was edifying, and the authors urge that it be included, even in models not based on thermodynamics, since it could account for apparent inhibitory interactions between species.

## 3. Modeling Approaches

This section describes the different types of modeling approaches to understanding the interactions within the microbiome and biofilms. The mathematical techniques and rules (or mechanisms) of these models are discussed. Most of the agent-based models described below have the microbes as autonomous agents which interact with each other and the environment. The models may use partial differential equations to describe the diffusion of the nutrients in the environment or within the biofilm/microbiome. Some of the models also use different time scales for different biological processes to reflect realistic situations. To speed up computations, parallel processing is often employed. Table 2 provides a summary of the models discussed in Section 3 with details about how the model was validated and what programming language was used.

### 3.1. Existing Software Platforms

#### 3.1.1. Netlogo

Netlogo is a generic agent-based simulation software that has been widely used for a variety of applications in fields as diverse as economics, environmental science, and sociology [74,84,85,86,87,88,89]. Netlogo provides a generic graphical user interface (GUI) environment that makes it easy to define ABMs. These models include the necessary agent types and their rules, as well as diffusible substances that exist in the same space, in addition to fluid flows. In Netlogo one can create sliders to easily alter parameter values such as initial bacterial population size, nutrient intake rates, flow rates, metabolite production rates, or parameters for switching mechanisms. The GUI also includes a map showing the location of the agents, a graph of the populations over time, reporting on the number and percentages of each agent group and level of nutrients. This real time visualization makes Netlogo models easy to understand and modify as needed for each application.

Netlogo and its variation GutLogo are popular options for developing biofilm and microbiome ABMs with three of the studies described thus far using them: the study by Weston et al. on the interaction of *Clostridia*, *Desulfovibrio*, and *Bifidobacteria* in the gut as a possible autism mechanism; the study by Lin et al. of four microbial genera within the inner wall of the ileum; and the study by Carvalho et al. on the role of switching between susceptible and persister phenotypes in antibiotic resistance of biofilms [28,37,46]. Specific rules in these models could easily be operated by Netlogo, such as the case in Lin et al. where rules were created to allow cells to adhere to the mucosal surface of the gut with a user defined probability [37]. This allowed cells to avoid washout due to the unidirectional fluid flow. Another example are the rules in Carvalho et al. that allowed cells to switch between susceptible and persister phenotypes according to several schemes: (a) randomly at a constant probability, (b) dependent on substrate concentration through a hyperbolic function, or (c) dependent on antibiotic concentration again through a hyperbolic function [28].

#### 3.1.2. iDynoMiCS

iDynoMiCS is an open-source software specific for simulations of biofilm growth, written in Java [47]. It defines a computational domain divided into the substratum, biofilm, boundary layer and the bulk liquid. The agents are the microbes with nutrients diffusing from the bulk through the biofilm. It allows for cells to grow and divide and to apply mechanical force to their neighbors (cell shoving). Due to its open-source nature, other researchers have created special versions to expand its capabilities. iDynoMiCS was used and modified to answer questions about the role that cell detachment, chemo-repellents or cellular aggregates play in the biofilm structure [34,35,43].

Li and colleagues added three detachment mechanisms to the iDynoMiCS software and used them to study the influence of cell detachment on *P. aeruginosa* biofilms [35]. The detachment mechanisms considered were fluid shear detachment, nutrient-limited detachment, and erosion which were dependent on biofilm thickness, nutrient concentration, and number of neighboring grid cells of an agent, respectively. To measure the biofilm growth, the authors employed average and maximum biofilm thickness, and biofilm cell numbers. To evaluate the morphology they estimated surface coverage, enlargement, and surface roughness.

Sweeney et al. modified iDynoMiCS to incorporate the production of quorum sensing, AI-2 molecules by the bacterial agents, diffusion of AI-2 through the environment, and introduction of planktonic cells [43]. Another significant addition was the possibility of a transition where some biofilm cells could become planktonic and vice versa. These transitions were controlled by crossing a threshold of chemotoxin concentration, which is user-defined. The authors also developed a new visualization tool to interpret the simulation results. The model is a good example of the use of agent-based simulations to examine the effect of chemotaxis on biofilm growth and structure. One limitation of the model is that it does not incorporate cell growth, division, and chemotoxin production by the planktonic cells.

Kragh et al. used iDynoMiCS to investigate how biofilm development can vary based on how it is seeded, either by single cells or larger cell aggregates [34]. The computer simulations were two-dimensional for computational efficiency. Oxygen was the limiting nutrient in Monod growth equations since they did not observe any changes in the experimental results when the concentration of the carbon source was changed. The initial aggregates were cut from a previously simulated biofilm and were then used to seed a new biofilm. The simulations ignored EPS production, detachment and cell-cell signaling.

This modeling platform was also used by Wright et al. to investigate whether biofilms favor damage segregation (DS), a fixed rate of repair (FR), or an adaptive repair (AR) [45]. The individuals in this model were “unicells” which were representative of unicellular prokaryotes and eukaryotes. Age was defined as a measure of the fraction of the biomass (or protein as referred in the paper) that is damaged due to metabolism. During growth, the cells converted the nutrient to protein which became damaged and inactive at a certain rate. Division was symmetric-both cells got half the damage, or asymmetric-old pole cell received all damage and new one got none. The repair strategies were none, FR and AR. Repair occurred when damaged material was converted into undamaged, active material at a loss of energy. Repair also led to shrinking. The authors incorporated AR by making the individual cells sensitive to their current intracellular damage levels and allocating an appropriate amount of newly synthesized protein for repair. This made the cells capable of responding to nutrient and stress gradients. FR allocated a fixed amount of protein to repair. AR was found to be the optimal strategy when the supply of substrate was limited, and when the rate of damage accumulation was proportional to the specific growth rate.

#### 3.1.3. LAAMPS (Large-Scale Atomic/Molecular Massively Parallel Simulator) (C++)

LAAMPS is an open-source software written in C++ distributed by Sandia National Laboratories, USA, which is primarily used for atomic and material modeling (https://lammps.sandia.gov/ Last accessed on 16 February 2021). The models by Jayathilake et al., Li et al., and Gogulancea et al. adapted LAAMPS to microbiology and created a biophysical agent-based model of biofilms, namely, the Newcastle University Frontiers in Engineering Biology (NUFEB) model [30,33,36]. All three models included biological, chemical, and mechanistic sub-models. These sub-models worked at different time scales. The mechanical forces within the models were contact force, cell-cell adhesion force, and fluid drag force. In all three models, the agents interacted with each other and the fluid.

A precursor of the NUFEB model, the mechanistic individual based model in Jayathilake et al. was a three-dimensional model with three functional groups and substrates [33]. The functional groups described above (HET, AOB, NOB) as well as their inactive counterparts, were considered. EPS was secreted by HET. The 3D model has a biological and physical sub-model. The former included Monod growth, division, shrinkage due to lack of nutrients and death, while the latter included adhesion between bacteria and EPS, contact between cells, and detachment. Additionally, fluid-bacteria interaction is included to simulate deformation and adhesion.

The NUFEB added fluid dynamics, pH dynamics, thermodynamics, and gas-liquid transfer to the Jayathilake et al. model [33,36]. NUFEB allowed for simulation of large complex microbial systems by dividing the entire domain into sub-domains, which allowed processing of each sub-domain with minimal interactions between them. It also included post-processing routines. Another innovation included the ability to model large numbers of organisms (up to 10^7^) through parallel computing and the addition of visualization and analysis routines. Another novelty of this model is with respect to the growth mechanisms. Growth of the bacterial agents depended on nutrient concentration, pH, and Gibbs free energy. The growth could be Monod based or energy based. If it was energy based, there was coupling with pH dynamics as well as gas-liquid transfer. Motion resulted from local mechanical interactions and fluid flow.

The agent-based model in Gogulancea et al., connected thermodynamics, pH, chemical speciation (influence of different forms of chemicals) and environmental conditions [30]. The scenarios modeled were aerobic and anaerobic conditions. The model classified the phenomena into three main categories: biological, chemical, and mechanical. Agents were spherical with their own parameters for division or becoming inert. The biomass yield was estimated using thermodynamics and the microbial growth was modeled using Monod equations. Metabolic networks for anabolism and catabolism were reduced to two main reactions. The growth expressions included the appropriate form of the chemical compound the bacteria could use for growth. The chemical module was for the transport and uptake of nutrients/excretion of metabolic products. The different processes modeled were diffusion of nutrients, reactor mass balance equations, gas-liquid mass transfer (important for wastewater treatment), and pH calculations. Mechanical interactions could describe attachment and detachment as well as pressure released when bacterial division occurs. Time steps for each process were user-defined. The code was also parallelized thus increasing speed of computations. The mechanical interactions employed a spatial domain decomposition strategy. For the biological and chemical processes, they decomposed the contents of the grid cells. The aerobic simulations were run under different boundary and pH conditions.

#### 3.1.4. Flux Balance Analysis with R

BacArena is an R package which includes multi-scale models of *P. aeruginosa* biofilms and a seven-species community of the human gut [26,90]. The R package is extendable, and computations can be executed in parallel. It incorporates flux balance analysis (FBA) with agent-based modeling and is based on the approach first developed for the MatNet model [91]. FBA is a reconstruction of the organism’s metabolism based on its genome annotation and subsequent calculation of the admissible steady state fluxes. In Bauer et al., the spatial environment was a 2-D grid [26]. Each agent had its own metabolism as well as followed biologically relevant rules like movement, duplication, chemotaxis, and lysis. In each time step, metabolites diffused in the environment and could be exchanged between the agents. The metabolism was modeled by the FBA of the species. The objective was to maximize biomass and the constraints were based on the metabolite concentrations. They validated the growth of the biofilm model with experimental data from literature. For the microbiome model, the seven species chosen were previously characterized experimentally [75]. For new R users, the line-by-line code and the fact that the package has multiple methods may be challenging. The lack of a robust GUI made the results less intuitive and easy to understand for a new user. However, the scaling was efficient, and plotting ranged from simple to complex with a lot of visual information.

#### 3.1.5. MASON Multi-Agent Simulation Toolkit

Tack et al. and Pérez-Rodríguez et al. utilized the multi-agent simulation toolkit MASON in Java to develop their agent-based models [39,44]. The model by Pérez-Rodríguez et al. investigated the effect of diffused N-acyl homoserine lactones (AHL) on the phenotypes within a *P. aeruginosa* and *C. albicans* biofilm [39]. Work by Tack et al. focused on the relationship between oxygen and the secretion of products by *E. coli* [44]. The authors developed a metabolic model for *E. coli* and incorporated it into MICRODIMS, (MICRObial Dynamics Individual-based Model and Simulator) [44,92]. The two case studies were 2D biofilm growth and a 3D submerged colony growth. The cell metabolism was incorporated using a linear model based on the intracellular metabolism in the form of FBA. The different processes included in the model were diffusion, update of local pH, nutrient and oxygen uptake, biomass growth, metabolite secretion, cell reproduction, cell lysis and movement. The cell movement included cell shoving and detachment. The different subprocesses were executed at different time steps. The simulations showed that oxygen limitations lead to local pH drops which in turn influence the biofilm population dynamics and detachment. The model is specific to *E. coli* and the authors caution that the model does not to apply to other species like *P. aeuroginosa*, though it could be adapted for food pathogens that are similar to *E. coli*.

AHL secreted by *P. aeruginosa* are known inhibitors of *C. albicans* hyphal development. In the model by Pérez-Rodríguez et al., AHL and *C. albicans* were modeled as spheres and *P. aeruginosa* as a rod [39]. AHL secreted by the bacteria diffuses within the biofilm until collision with the fungus or the boundary. The diffusion was based on viscosity of the medium and radius of the spherical particles. The simulations studied the impact of spatial location of the fungal cells with respect to the bacteria, the impact of number, orientation, and localization of cells over cell communication, influence of AHL molecules produced outside the vicinity, and finally the interactions within a biofilm. The biofilm model was seeded with 2 fungal and 5 bacterial cells which were randomly distributed within the environment.

### 3.2. Other Modeling Strategies

The ABMs described in Section 3.2 have a variety of interesting features such as inclusion of intracellular signaling, rod shape for the bacterial agents, inclusion of eDNA strands, and using bacterial aggregates to seed a biofilm. However, these models are encoded in software that is problem-specific, and therefore difficult to extend to other problems.

A software tool called Cell Modeler was created to design synthetic biofilms for industrial use [40]. The ABM was 3-dimensional and could include many cell properties, including genetic regulation and intracellular signaling. The model employed different time scales for different processes. Most of the rules as well as the growth function could be user-defined. They validated their model using live cell culture and fluorescent microscopy methods.

The model of bacterial biofilm growth by Jin et al. was a mix of the traditional agent- based model and differential equations models [93]. The bacterial agents absorbed substrates (nutrients, minerals, and other chemicals) and water and used them for growth, reproduction, and EPS production. The EPS and water were modeled as interacting continua using partial differential equations. Interactions between bacterial cells and cell-to-wall were modeled using a discrete element method. The model was written using FORTRAN and, unfortunately, no link to the software was provided.

The authors of Hartmann et al. investigated *V. cholerae* biofilms with emphasis on the influence of external fluid flow on the structure and morphology of the biofilms [31]. This model has both a continuum and an agent-based component. In the ABM, the agents were ellipsoids which grew and divided but were devoid of self-propulsion. The agents were described by their position and orientation. There was a force of attraction and repulsion between the agents and the wall boundary. The code could also be run in parallel to reduce computational load. All the parameters were determined from experimental biofilms and validation was done with parameters associated with the phenotypes and morphology.

Das et al., employed statistical physics data analysis techniques with computational analysis tools to study *H. influenzae* biofilms formed in the middle ear [29]. The authors focused on the influence of the eDNA network and Tfp expression on biofilm structure. The agent-based model is two dimensional with the simulation box divided into compartments. The agents are the biofilm as well as planktonic cells, eDNA strands and nutrients. The agents grew using a kinetic Monte Carlo scheme and replicated within a single compartment. They moved to another compartment when a threshold was reached. The biofilm cells could also disperse into the liquid medium. The eDNA strands were produced by the bacterial cells at a fixed rate. These strands formed a network within the substrate and could diffuse into neighboring compartments. In the model, the eDNA network facilitated movement of the cells between compartments. At constant intervals, nutrient was introduced into the system and several planktonic cells were removed. All the model parameters were obtained from the literature.

The agent-based model by Head et al., has bacterial agents which were all assumed to be acidogenic, i.e., capable of producing acid through glycolysis [32]. The population was divided into aciduric (capable of metabolizing glucose to acid in a low pH environment) and non-aciduric. The rectangular domain was divided into an enamel surface, biofilm, and the saliva. The initial seed for the biofilm were cell aggregates. The biofilm cells grew, produced EPS, and divided based on the glucose concentration and the pH. The cells also were removed once a specified height was reached. The glucose was the sugar which diffused through the environment. The authors chose glucose because of the large amount of published data on glycolysis.

The agent-based model described by Beroz et al., was written in C++ and incorporated rod-shaped cells which grew, divided, adhered to the surface, and had cell-cell, and cell-surface interactions [27]. The model investigated the phenomenon of verticalization (cells have vertical orientation to the surface) observed in experimental biofilms. They employed external forces on the cell to observe if there was instability to vertical reorientation. When the pressure exerted by the external force crossed a specified threshold, the cell became unstable to spontaneous reorientation. They observed that verticalization depended on cell length and began when the surface pressures due to growth overcame adhesive forces. The authors also employed a 2D continuum model using Python to better understand the relation between local verticalization and global dynamics of the biofilm.

The Java agent-based model by Shashkova et al., of the microbiome was comprised of two bacterial species, metabolites, and the gut; all of these were represented as autonomous agents [42]. The bacterial agents were capable of inter-species interaction as well as interaction with the gut. The bacteria were governed by metabolite and gut interactions as well as the number of objects (metabolites and bacteria). The life cycle of the bacterium was to produce metabolites, search for nutrients, divide, or die. The metabolites only moved through the gut and were either absorbed by the bacterial agents or the gut wall. They could also be excreted by the gut wall. The excretion controlled the abundance of the metabolites and thus directly influenced their flux. The gut was divided into 3 areas, namely, the mucin layer, lumen, and border between the two. The kinetics were modeled using ordinary differential equations. The authors investigated the effect of antibiotic treatment on the model. The scenarios tested were one or both of the bacteria being sensitive to the antibiotic, or both being resistant. The model also incorporated a probability of antibiotic resistance mutation.

The Simbiotics platform is a multicellular model with bacterial agents which interact with each other and the environment [38]. It is a hybrid model that combined an agent-based model of bacteria with a continuous chemical environment. The environment has gradients of the nutrients. Cells may be spherical or rod-shaped and their local environment has both information about their neighbors as well as the chemical environment. Motion of cells was determined by Newtonian mechanics with force due to cell collisions, adhesion, electrostatic interactions, diffusion, drag and force of gravity. The biological processes implemented were growth, metabolism, division, movement, quorum sensing through membrane transport, adhesion as well as gene regulatory networks. Intracellular processes were discrete in the form of Boolean networks or differential equations. The software has several customizable submodels for different processes. One could also use Z-stack images to initialize the simulation. All the case studies in the paper were based on experiments. The authors also developed Easybiotics, a user-friendly version of Simbiotics [94]. It added live graph-plotting and parameter sweeps to the output [94]. A visual representation of the modular code was incorporated to allow for easier compiling.

## 4. Discussion

Flemming et al., described biofilms as one of the most ubiquitous and successful modes of life on Earth, which also affect several biological processes in mammalian hosts [95]. Important polymicrobial interactions occur on skin and mucosal surfaces which contain bacteria, archaea, and eukaryotes [4]. There is a strong effort by several large research consortia such as MetaHIT, the Human Microbiome Project and American Gut to profile the human microbiome in health and various disease states [96,97,98]. To better understand the vast amount of data generated by these consortia, developing multi-species modeling approaches becomes imperative. These approaches can lead to innovative, experimentally testable hypotheses.

Our review focused on the agent-based modeling approaches adapted by various research groups to understand interactions of microbial cells with each other and with their environment while growing in a sessile or planktonic state. Their work explored a multitude of different biological questions such as antibiotic resistance, control of medically important biofilms, the microbiome, and industrial uses of biofilms [25,26,28,30,31,33,34,35,36,37,42,43,46,93].

Some models included interactions between agents and the environment. The environment in most of the models included the gut (for microbiome models) or a surface of attachment (for biofilm models) with metabolites that diffuse with the help of differential equations. Several models included multiple time scales and parallel computing to speed up computation [30,33,36,40,44]. Some of the models incorporated complex programming concepts which would be daunting to a novice programmer and harder to modify [26,27,29,31,93]. Biological parameters in models are most often defined using data from prior experimental studies, although flux balance analysis, based on organism genome annotations, is a recently-introduced alternative to laboratory data [26,44]. Many modelers have used existing experimental data to validate their results [26,30,32,33,35,36,39,40,42,44,46]. For example, cooperative metabolic phenotypes that were predicted by BacArena for *P. aeruginosa* biofilms were previously discovered experimentally in *Bacillus subtilis* biofilms [26,99]. Similarly, the model created by Tack, et al. predicted mushroom-shaped structures and the formation of chains of *E. coli* cells at the exterior biofilm surface under flow, as seen in earlier experimental studies [44,100,101]. The most comprehensive studies started by building models, and making novel predictions, which were subsequently validated experimentally [25,27,29,31,34,38,41,43]. Some of these experiments involved generation of new mutant strains, i.e., a *V. cholerae* mutant deficient in the production of EPS was used to test predictions about the advantages of adhesion in biofilm formation [41]. Other researchers used physical or chemical changes to the agents or their environment to validate predictions. For example, after their model predicted that diffusion of bacterial cells was a major determinant of biofilm structure, Acemel et al. used dextran to increase media viscosity, slow diffusion of bacteria, and reproduce the predicted structure [25]. As ABMs become more complex, incorporating more species and more varied interactions with their environment, it will be increasingly important to ensure that they are accurate representations of biological systems. This can best be accomplished by close collaboration and scientific exchange of experimental data and modeling tools between mathematicians and microbiologists. However, one of the powerful contributions of mathematical modeling in studying highly diverse microbial communities is producing a level of complexity that cannot always be recapitulated experimentally.

Obviously, the applications that were developed with open and widely available software platforms (NetLogo, iDynoMiCS, LAAMPS, MASON, and R) are easier to reproduce than those that were based on special-purpose software. In our search of the literature the latter were mostly unavailable and likely harder to adapt to other applications, if one so desired. This has consequences for reproducibility as can already be inferred from the several studies which built upon existing models based on open-source software applications [102,103,104]. These open-source frameworks produce a base that can be modified (e.g., expanding iDynoMiCs to include detachment and planktonic cells) and help propel the field forward [35,43].

Agent-based modeling of biofilms and microbiomes is clearly becoming a popular way to study microbial interactions, in part because of the accessibility of model construction to domain experts with limited modeling expertise. We encourage biologists to consider adopting one of several open-source and user-friendly modeling platforms as part of their cycle of hypothesis generation and testing [26,37,38,46,47,94]. The trend appears increasingly to be the use of models containing a limited number of species and focusing on their interactions. Increasing the capabilities of these models to include a higher number of functionally different microbial agents is needed so that they can be used to model the complex microbiota harbored in the oral-digestive tract, and their interactions. A development that is expanding the scope of research questions is the inclusion of specific shapes for the microbial agents. Whereas older biofilm ABMs were restricted to agents having spherical shapes, we are now seeing the appearance of studies with other shapes, such as rods. We expect this trend to expand to other relevant shapes, such as the pleiomorphic shapes of certain commensal and pathogenic fungi. In the future we expect to see more studies using agent-based modeling of diverse polymicrobial communities as tools to generate novel hypotheses that can be followed by appropriate experimental validation.

## Figures and Tables

**Table 1:** Summary of models with information about the interactions represented within the model.

Reference	Environment		Interactions						
	Microbiome	Biofilm	Physical	Biological	Microbe/Host	Metabolic	Microbe/EPS	Toxin/Antitoxin	Chemotaxis
Acemel et al., 2018 [25]		X	X	X					
Bauer et al., 2017 [26]	X	X	X	X		X			
Beroz et al., 2018 [27]		X	X	X		X			
Carvalho et al., 2018 [28]		X	X			X			
Das et al., 2017 [29]		X	X	X		X			
Gogulancea et al., 2019 [30]		X	X	X		X	X		
Hartmann et al., 2019 [31]		X	X				X		
Head et al., 2017 [32]		X	X	X		X			
Jayathilake et al., 2017 [33]		X	X	X		X	X		
Kragh et al., 2016 [34]		X	X	X		X	X		
Li et al., 2015 [35]		X	X			X	X		
Li et al., 2019 [36]		X	X	X		X	X		
Lin et al., 2018 [37]	X				X	X			
Naylor et al., 2017 [38]		X	X	X					
Pérez-Rodríguez et al., 2018 [39]		X	X	X		X			X
Rudge et al., 2012 [40]	X	X	X	X					
Schluter et al., 2015 [41]		X	X	X		X	X		
Shashkova et al., 2016 [42]	X				X	X		X	
Sweeney et al., 2019 [43]		X	X	X		X	X		X
Tack et al., 2017 [44]		X				X			
Wright et al., 2020 [45]		X	X	X		X	X		
Weston et al., 2015 [46]	X				X	X		X	

**Table 2:** Summary of models with information about the programming language, validation and other model characteristics. Links to models are included. All links were last accessed on 16 February 2021.

Reference	Environment		Characteristics			Validation Method			Programming Language
	Microbiome	Biofilm	2D	3D	Parallel	Experimental Data, This Study	Experimental Data, Literature	None	Click Links to Software if Available
Bauer et al., 2017 (BacArena)	X	X	X				X		R
Beroz et al., 2018		X	X			X	X		C++
Carvalho et al., 2018		X	X					X	NetLogo *
Das et al., 2017		X	X			X	X		not stated
Gogulancea et al., 2019 (NUFEB)		X		X	X		X		C++-LAMMPS
Hartmann et al., 2019		X	X		X	X			not stated
Head et al., 2017		X	X				X		not stated
Jayathilake et al., 2017 (NUFEB)		X		X	X		X		C++-LAMMPS
Jin et al., 2020		X	X					X	Fortran
Kragh et al., 2016		X	X	X		X			Java **
Li et al., 2015		X	X	X			X		Java **
Li et al., 2019 (NUFEB)		X		X	X		X		C++-LAMMPS
Lin et al., 2018	X		X					X	Netlogo
Naylor et al., 2017 (Simbiotics)		X	X	X	X	X	X		Java
Pérez-Rodríguez et al., 2018		X	X				X		Java ***
Rudge et al., 2012 (Cell Modeler)	X	X		X			X		Python
Shashkova et al., 2016	X				X		X		Java, R
Sweeney et al., 2019.		X	X	X		X			Java **
Tack et al., 2017		X					X		Java ***
Wright et al., 2020		X	X	X				X	Java **
Weston et al., 2015	X				X		X		Netlogo

* Source code is available under ‘Bacterial persistence in biofilms’ ** Modifications to the iDynoMiCS code *** Based on MASON toolkit.

## Data Availability

No additional data.
